# Aldo van Eyck’s Playgrounds: Aesthetics, Affordances, and Creativity

**DOI:** 10.3389/fpsyg.2017.01130

**Published:** 2017-07-04

**Authors:** Rob Withagen, Simone R. Caljouw

**Affiliations:** Center for Human Movement Sciences, University Medical Center Groningen, University of GroningenGroningen, Netherlands

**Keywords:** aesthetics, affordances, Aldo van Eyck, children, creativity, playgrounds, standardization

## Abstract

After World War II, the Dutch architect Aldo van Eyck developed hundreds of playgrounds in the city of Amsterdam. These public playgrounds were located in parks, squares, and derelict sites, and consisted of minimalistic aesthetic play equipment that was supposed to stimulate the creativity of children. Over the last decades, these playgrounds have been studied by sociologists, theorists of art and architecture, and psychologists. Adopting an ecological approach to the human environment, it is argued that the abstract forms of van Eyck’s play sculptures indeed stimulate the creativity of the child. Whereas a slide or a swing almost dictates what a child is supposed to do, van Eyck’s play equipment invites the child to actively explore the numerous affordances (action possibilities) it provided. However, it is argued that the standardization (e.g., equal distances between blocks or bars) that tends to characterize van Eyck’ play equipment has negative effects on the playability. This standardization, which was arguably the result of the aesthetic motives of the designer, might be appealing to children when simply looking at the equipment, but it is not of overriding importance to them when playing in it. Indeed, a recent study indicates that the affordances provided by messy structures appear to have a greater appeal to playing children.

## Introduction

“We must understand that art and life are no longer separate domains. The idea that art is *an illusion divorced* from real life must therefore be abandoned. The word ‘Art’ means nothing to us. We demand that it be replaced by the *construction of our environment according to creative laws* derived from well-defined principles.” (van Doesburg and van Eesteren, 1923; included in Baljeu, 1974, p. 147).

Between 1947 and 1978, the architect Aldo van Eyck was involved in designing hundreds of playgrounds in the city of Amsterdam. Although only 17 of his playgrounds are left today (van Lingen and Kollarova, 2016), van Eyck’s project continues to have an impact on thinking about cities, architecture, playgrounds, and children. Over the last two decades, the playgrounds of van Eyck have been honored and studied by different academic disciplines, including sociology, art, architecture, and psychology (e.g., Lefaivre and Tzonis, 1999; Fuchs, 2002; Strauven, 2002; Solomon, 2005, 2014; Sennett, 2008; Jongeneel et al., 2015; van Lingen and Kollarova, 2016; Sporrel et al., 2017; Sporrel et al., unpublished). In the present paper, we will analyze van Eyck’s playgrounds drawing upon these diverse disciplines.

We will start with a portrayal of van Eyck’s playscapes in Amsterdam, emphasizing certain aspects of them and linking them to the architectural theory of the “humanist rebel,” as van Eyck has been called (Lefaivre and Tzonis, 1999). Then, we will sketch in bold strokes an ecological approach to the human environment. This approach, which was initiated by the psychologist Gibson (1979) in the 1960s and 1970s, provides a framework for understanding the environment we live in. Moreover, this framework can elucidate some insights from the disciplines of art, architecture, and sociology. In particular we will focus on two aspects of van Eyck’s playgrounds: the “open function” of his play equipment that is supposed to stimulate the creativity of the child; and the standardization of his equipment that gives it an aesthetic appeal but might have negative effects on the playability.

## Van Eyck’s Playgrounds

In 1946, one year after World War II came to an end in the Netherlands, Aldo van Eyck was appointed as an architectural designer in the town planning section of the Amsterdam public works department. Among his first tasks at this department was to design a public playground at the Bertelmanplein in Amsterdam. For this square van Eyck designed a climbing arch, three tumbling bars, and a rectangular sandpit with a rim that is lowered at two places to let small children enter it. Moreover, a couple of benches were placed at the square, allowing parents to look after their playing children (e.g., Solomon, 2005; van Lingen and Kollarova, 2016).

The town planning section of the city of Amsterdam wanted to have a playground in each neighborhood in Amsterdam, a city of which parts were destroyed during the war. Although van Eyck stopped working at the public works department after 5 years (to become a lecturer in art history and start his own company), he continued working on his playgrounds. In the period between 1947 and 1978, he designed no less than 734 playgrounds in Amsterdam. Indeed, “the project took the city by storm” (Lefaivre and Tzonis, 1999, p. 17). Besides the impact that van Eyck’s playgrounds had on the social life in Amsterdam, they were also of great architectural significance. As the theorists of architecture Lefaivre and Tzonis (1999) put it,

Even if for some reason van Eyck had not designed anything but this galaxy of playgrounds, if he had not achieved anything beyond this attempt to implant a ‘starry sky’ of over seven hundred playgrounds in postwar Amsterdam, his place among the major figures of architecture of this century would have been secured. It is here that the major breakthroughs of an architecture of ‘community’ and ‘dialog’ and of the human and formal building of the ‘realm of the inbetween’ take place. (p. 77).

Aldo van Eyck was rebelling against the program of CIAM (Congrès Internationaux d’Architecture Moderne), which was founded in 1928 and flourished in the 1930s and 1940s. In the *Athens Charter*, le Corbusier opted for a massive rebuilding of cities in which the functions of labor, living, and leisure are spatially segregated, and street life was reduced to traffic flows (e.g., Frampton, 1980). Although Aldo van Eyck attended several meetings, he severely criticized CIAM’s practices, and tried to replace it with a humane architecture.

Van Eyck was very much inspired by Buber’s (1923) seminal book *Ich und Du*, a manuscript that he started studying while he was student in Zurich. Among other ideas, van Eyck adopted Buber’s contention that dialog is foundational for life.

There is only one reality between real persons – what Buber call ‘the real third’. […]

The real third is a real dialog, a real embrace, a real duel between real people.

   Buber then goes on to state – and this is his crucial point – that the real third is not something that happens to one person or another person separately and a neutral world containing all things, but something that happens in a dimension only accessible to both. The in-between acquiring form (van Eyck, 1962/2008, p. 54).

Van Eyck developed an architecture of the *in-between realm*—“Its job is to provide this in-between realm by means of construction, i.e., to provide, from house to city scale, a bunch of real places for real people and real things” (van Eyck, 1962/2008, p. 55). In developing his theory, van Eyck emphasized that concepts are needed that have a bearing on the daily life of people. “Space has no room and time is not a moment for man. He is excluded” (van Eyck, 1960/2008, p. 471). In his view the abstract concepts of space and time need to be replaced by place and occasion, concepts that “include him” and thus “mean more.”

Aldo van Eyck’s humane architecture aimed at creating places that fostered dialog and stimulated community life in which children take part. Ever since the 16th century, children’s play has been important in Dutch culture—several paintings from this century onwards have children playing as their main topic (e.g., Schama, 1987; Lefaivre and Tzonis, 1999), and the historian Huizinga (1938) celebrated play in his landmark book *Homo Ludens*. Hence, with his emphasis on children and their playing Aldo van Eyck stood in a long tradition. However, the goal he had was ambitious. As van Eyck put it poetically:

To consider the city is to encounter ourselves.

To encounter the city is to rediscover the child.

If the child rediscovers the city,

the city will rediscover the child – ourselves.

LOOK SNOW!

A miraculous trick of the skies – a fleeting correction.

All at once the child is Lord of the City.

But the joy of gathering snow off paralyzed vehicles is short-lived.

Provide something for the human child more permanent than snow – if perhaps less abundant.

Another miracle.

van Eyck (1962/2008, p. 25).

Although the importance of leisure and children’s play was recognized by members of CIAM, they aimed to realize it in a completely different way from van Eyck. Le Corbusier, for example, imagined leisure in “idealized settings,” often at a serious distance from the houses of the children (Lefaivre and Tzonis, 1999, p. 51). Van Eyck, on the other hand, designed and created playscapes in the neighborhoods of an already existing city, accepting and taking advantage of all the constraints that come with it. Indeed, he followed Theo van Doesburg’s dictum that all parts of the city are of equal importance and should be used. Thus, contrary to the program of CIAM that pleaded for a massive rebuilding of the city, van Eyck adopted an “infill” strategy, using existing and ignored spots in the city to create places for social gathering and children’s play (Lefaivre and Tzonis, 1999; Solomon, 2005). During WO II, many houses were destroyed and there were thus ample derelict sites that could serve this purpose. Encouraged by the public works department and the citizens of Amsterdam (who explicitly asked for a playground in their neighborhood), van Eyck ended up designing more than 700 site-specific playgrounds. There are two aspects of these playgrounds that we would like to emphasize.

### Merging into the City

**FIGURE 1:**
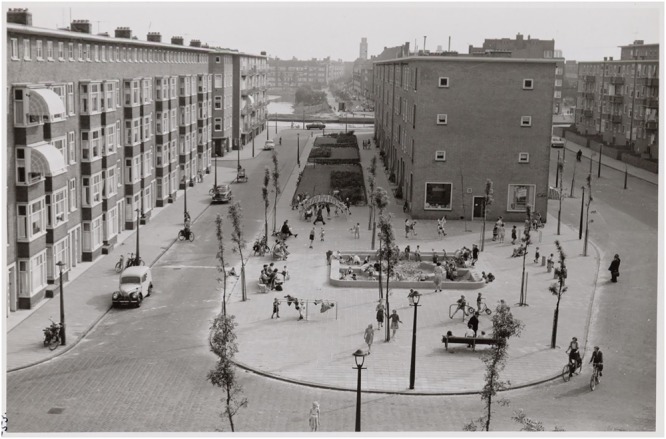
Aldo van Eyck’s playground at the Buskenblaserstraat in Amsterdam. (Courtesy of the Amsterdam City Archive; reprinted with permission).

One characteristic of van Eyck’s playgrounds is that, although located in a city, they were never fenced. This was rather exceptional in the 1950s and 1960s. At that time the contrived playgrounds were generally isolated places—they were surrounded by a fence with a gate, and children had to pay a little fee (or be a member) to enter it. Often a guard was appointed who was responsible for the supervision of the children. Van Eyck, on the other hand, strived at merging the playgrounds with the city. The playground he created at the Buskenblaserstraat in Amsterdam provides a nice illustration of this (**Figure 1**). There were no sharp boundaries that separated the playground from the rest of the city. The sociologist Sennett (2008) emphasized this aspect and the role it could have for children’s play.

Van Eyck intuited that such spatial ambiguities would also provoke children to engage with one another, toddlers tending to help each other crawl and totter about. This intuition was elaborated in the making of the Buskenblaserstraat park. Here a park was contrived from empty space at a street corner, with cars flowing past. While the sandpit here is well marked and set well back from the streets; equipment for children to climb on has not been so protected. Cooperative activity—looking out for cars, shouting, lots of shouting—becomes a matter of keeping safe […]. And just because in the Buskenblaserstraat there is enough room for tossing and kicking balls around, kids have had to come up with game rules that permit play without their being hit by cars. The architect, then, designed a park using the simplest, clearest elements that invite its young users to develop the skill of anticipating danger and managing it; he did not seek to protect them through isolation (p. 233).

By not fencing the playing children, they became an integral part of the city. Moreover, by placing benches at the square, van Eyck created a place that invited parents or guardians to oversee their children and to gather together. Street life and community were stimulated (e.g., Solomon, 2005).

van Lingen and Kollarova (2016) recently argued that van Eyck also established the integration of the playgrounds into the city by two other means. First, van Eyck created play elements using mainly metal and concrete. Contrary to the brightly colored plastic that is so popular today in playground design, these materials fit in naturally with the building materials of the city. Second, an “urban character” (van Lingen and Kollarova, 2016, p. 68) of the play elements was realized by the use of elementary forms (Strauven, 2002), a topic to which we shall now turn.

### The Aesthetic, Minimalistic Play Elements

**FIGURE 2:**
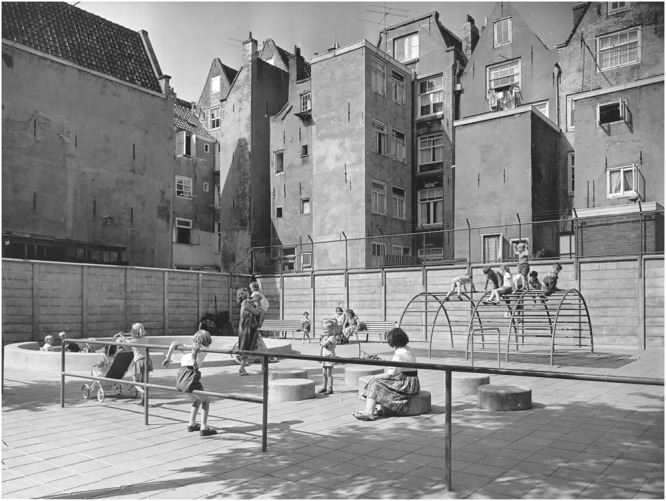
Aldo van Eyck’s playground at the Laagte Kadijk in Amsterdam. (Courtesy of the Amsterdam City Archive; reprinted with permission).

**FIGURE 3:**
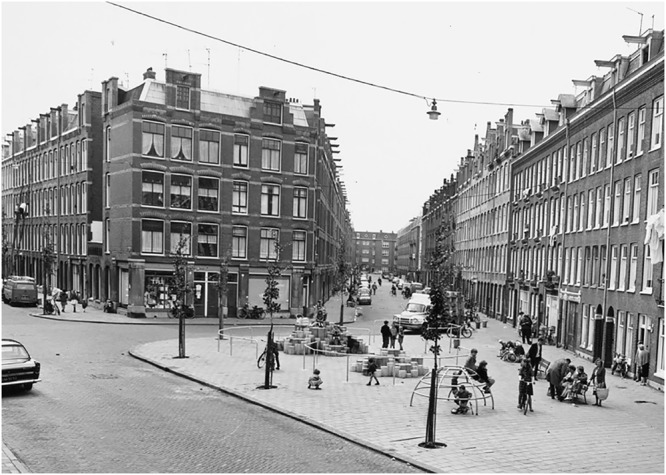
Aldo van Eyck’s playground at the Van Boetzelaerstraat in Amsterdam. (Courtesy of the Amsterdam City Archive; reprinted with permission).

As mentioned earlier, van Eyck created playgrounds in existing parks, squares, and other empty places in the city, taking into account the constraints that were provided by these places. Consequently, each playground was site-specific and unique. However, van Eyck created a set of play elements that he used and harmoniously combined in the design of the different playgrounds. Among these play elements are the above-mentioned sandpit, climbing arch, and tumbling bars that were placed in his first playground. Later he also designed a popular and widely copied climbing dome, jumping stones, and a climbing mountain (**Figures 2, 3**). A characteristic of many of van Eyck’s play elements is that they are often geometrical, giving them an aesthetical appeal. As Lefaivre and Tzonis (1999) put it, “[l]ooking at the configuration out of which the playgrounds are made, we are immediately struck by the predominance of clear geometrical shapes: circles, squares, and triangles” (p. 70).

Van Eyck was in touch with and inspired by Constantin Brancusi. He dedicated his book *The child, the city, and the artist* (1962/2008) to the Romanian sculptor, and included a quote from him: “[s]implicity is not a goal in art but one reaches simplicity in spite of oneself, by approaching the real sense of things” (quoted in van Eyck, 1962/2008, p. 30). In his search for the essence of things, Brancusi generally ended up with abstract, powerful geometrical shapes. His well-known sculpture *The Kiss* offers a case in point. Although the kiss has been a theme in Western art for centuries and has been represented in all its detail and gracefulness, Brancusi aimed to capture its intensity by “a masterful elimination of anatomical features” (Geist, 1978, p. 12). Even in the first versions of *The Kiss*, which are less abstract than the later versions, there are no noses, ears, elbows, chins, and throats. And this elimination gives the sculpture its impact—“[f]ree of impedimenta, the image speeds to eye, invades the mind” (Geist, 1978, p. 13).

Brancusi’s sculptures have influenced van Eyck in the design of his play elements (Strauven, 2002). Indeed, one can conceive of these elements as sculptures in the tradition of Brancusi—abstract, minimalistic forms that similarly “invade the mind.” In the remainder of this paper, we will analyze these play elements from an ecological approach to the human environment.

## An Ecological Approach to the Human Environment

In the 1960s and 1970s, the American psychologist James Gibson developed an ecological approach to psychology. This approach aimed to understand how animals, including human-beings, perceive and act in their environment. As Gibson (1979) started his landmark book *The ecological approach to visual perception*,

This is a book about how we see. How do we see the environment around us? How do we see its surfaces, their layout, and their colors and textures? How do we see where we are in the environment? How do we see whether we are moving and, if we are, where we are going? How do we see what things are good for? How do we see how to do things, to thread a needle or drive an automobile? Why do things look as they do? (p. 1).

Gibson argued against psychologies that do not do justice to lived experience and everyday behavior. Like van Eyck, he criticized the concepts of space and time, notions that psychology had adopted from classical physics and that held it captive for centuries. At the time Gibson developed his ecological approach, cognitive psychology was in its ascendancy. This psychology started from the physicalist assumption that the environment is meaningless, consisting solely of matter in motion. To understand how we experience a meaningful environment (full of color, smell, taste, and so on), cognitive psychology claimed that in the process of perception our brain creates a perceived world—it attaches meaning to the stimulus information that our senses receive. Gibson believed that this approach is misguided from the very start—the assumption that “we live in a physical world consisting of bodies in space […] is very dubious” (p. 16). Indeed, in his view concepts like space and time are “ghosts” (Gibson, 1975, p. 295), they have no bearing on the everyday behavior of humans (see also van Dijk and Withagen, 2016).

In the autumn of his life, Gibson developed an alternative theoretical framework, focusing on the animal, the environment, and their relationship at an ecological scale. A central tenet of Gibson’s ecological approach is that the environment we live in does not consist of matter in motion in space; rather it consists of possibilities for action. He coined these possibilities *affordances*, and defined them as follows.

The *affordances* of the environment are what it *offers* the animal, what it *provides* or *furnishes*, either for good or for ill. The verb *to afford* is found in the dictionary; but the noun *affordances* is not. I have made it up (p. 127; italics in original).

For example, for a human-being a chair affords sitting, a floor affords walking upon, water affords drinking, and so on. There are two aspects of the affordance concept that need to be emphasized here. First, affordances exist by virtue of a relationship between the properties of the environment and the action capabilities of the animal. Whether a glass affords grasping with one hand depends on the size of the cup relative to the span and flexibility of the hand—a cup that might be graspable for an adult might not be graspable for a toddler. Hence, to determine the affordances of the environment for an animal, we have to measure the environment not in terms of metric units (i.e., meters), but in terms of the animal’s action capabilities. Thus, an affordances-based description of the environment “includes” the animal (Costall, 1999, 2004). Second, and related to this, describing the environment in terms of the affordances of an animal points to the *functional significance* this environment has for the animal. It refers to what the animal can do in his environment, what it means to him (Gibson, 1982).

Ever since its introduction, the concept of affordances has proven to be useful to understand the environment and our behavior in it (e.g., Kyttä, 2002, 2004; Rietveld and Kiverstein, 2014; Cordovil et al., 2015; Prieske et al., 2015; Menatti and Casado da Rocha, 2016; Withagen and Caljouw, 2016). In his study of the environment of children, Heft (1988), for example, contrasted an affordance-based description of the environment with a “form-based classification of environmental features” (p. 29). The latter refers to our everyday description of our environment. When describing a park, for example, we mention a tree that is in the middle of a grass court, the lake, and the benches at its side. Heft claimed that such a form-based description considers the properties of the environment to be independent of the individuals who use them, and, thus, “provide little insight into the functional, and hence, the psychological significance of environmental features” (p. 36). An affordance-based description of the environment, on the other hand, is relative to the user and puts the functional significance of the environment center stage. Moreover, contrary to a form-based description, an affordance-oriented one recognizes that a single object can have different meanings to an individual. As Gibson (1979) had already emphasized, a single object can afford different behaviors to an animal. For example, a child can sit on a bench, but can also step on it, and jump from it.

Also in the context of architecture the concept of affordances has proved its mettle, both in the analysis of the built environment and in the design of it. Beek and de Wit (1993), for instance, adopted this concept in analyzing the City Orphanage in Amsterdam, another celebrated project of Aldo van Eyck. Besides paying attention to affordances at the scale of the individual (e.g., a chair affords sitting, a floor affords running), Beek and de Wit (1993) also emphasized what they called the “social and socioeconomic affordances” (p. 36). Aiming at fostering social behavior and communication, van Eyck created many little squares and meeting places in his building that afforded (and invited) children to play together or to meet with the caregivers. A studio that takes the concept of affordances central in the design process is RAAAF (Rietveld Architecture Art Affordances). They conceive of “architectural interventions” in the environment as the construction of affordances (Rietveld and Rietveld, 2011). Among other things, they recently created an office of the future which they coined “The end of sitting.” Being inspired by a newspaper article mentioning the detrimental health effects of prolonged sitting, they created a sculpture consisting of slanted surfaces that afford working in several non-sitting postures like leaning and (supported) standing (e.g., Rietveld, 2016; Withagen and Caljouw, 2016).

### Stimulating Creativity

The above affordance perspective can help in elucidating some insights from theorists of art and architecture. These theorists have often valued the abstract, simple forms that van Eyck used because it leads to play elements with an “open function.” As the historian of art, and former director of the Stedelijk Museum in Amsterdam, Fuchs (2002) put it,

The playgrounds were fantastic because the objects were simple: rectangular and round frames for climbing (the latter like an igloo), a sandpit, a group of circular concrete blocks for jumping from one to the other – objects that are not anything in themselves, but which have an open function and therefore stimulate a child’s imagination. A child sits still on a slide or a swing: it is the object that produces the movement. Van Eyck’s objects do not move, but they allow a child to move with all the acrobatism and suppleness he can muster. That was the genius of their simplicity (p. 7).

Studying the many pictures of van Eyck’ playgrounds and visiting the remaining playgrounds ourselves, indeed suggest that children use many of the affordances that the play elements provide them. The rim of the sandpit is used by the children to climb on, to jump over, to run upon, and also provided a work surface while they are playing with the sand. Moreover, many parents use the rim as a thing to sit on while looking after their children. Another example is the “climbing” dome (**Figure 3**). This element that van Eyck designed in 1957 was rather popular in the decades that followed and is now placed in the garden of the Rijksmuseum in Amsterdam. Children climb on this dome, but also sit on top of it, jump from it, and use it as a little house to dwell in and to gather together. van Lingen and Kollarova (2016) mentioned that some women also used these domes to beat their carpets on.

However, and as mentioned above, Gibson had claimed that nearly every object affords different activities for an individual. This holds true also for conventional play elements like a slide and a see-saw. Children can use a slide as a thing to climb on (using the ladder) and slide down, but they can also jump from it after climbing to the top (if the slide is not too high). Or they can climb to the top via the sliding part using their hand and feet. However, the fact that these conventional play elements are generally used in a single way whereas van Eyck’s elements are often used in multiple ways can be elucidated from a sociocultural perspective on affordances.

This perspective was initiated in the 1980s and 1990s by a number of authors (e.g., Heft, 1989, 2001; Hodges and Baron, 1992; Costall, 1995; Reed, 1996; Ingold, 2000; for some recent developments see, Rietveld, 2008; Rietveld and Kiverstein, 2014; van Dijk and Withagen, 2016; van Dijk and Rietveld, 2017). A central tenet of this approach is that the use of objects (and their affordances) always takes place in and is largely shaped by the sociocultural environment. For example, we learn about the affordances of objects from and through other people. To elucidate this point, Costall (1995) quoted Leont’ev (1981).

[The] notion of an individual, a child, who is all by itself with the world of objects is a completely artificial abstraction. The individual is not simply thrown into the human world; it is introduced into this world by the people around it, and they guide it in that world (p. 135).

A child entering a playground perceives other children using the equipment and/or is introduced to it by the parents. Especially in the case of young children, parents guide their child to, for example, the slide, supports it while she climbs the ladder, and encourages her to slide down. By doing so, the parents demonstrate the child *the* function of the play element. Costall (2015) called such a function the “canonical affordance” of the object, to refer to its “single, definitive meaning” (p. 51; see also Costall, 2012) within a social practice. Indeed, when a child uses the slide in another way (e.g., by climbing up via the part that is meant to slide down), many parents correct their children that this is not how they should use the equipment—this is not “what the object was made for” (see also Kyttä, 2004, on “the field of constrained action”).

Van Eyck intentionally created play equipment that does not have such a “single, definitive meaning.” He conceived of his abstract play elements as “tools for imagination,” to use his own phrasing (de Roode, 2002). As van Lingen and Kollarova (2016) put it,

One of the most important aspects of the play elements van Eyck designed is that they do not have a designated function: They can be used in different ways, depending on the game you are playing, and with their simple and abstract forms they stimulate children to use their imagination[…]. Aldo van Eyck’s designs don’t aim to show what they are and how they should be used, they rather suggest what they could be (p. 48).

If we consider children playing on, say, the above-mentioned dome, it is indeed hard to conceive of an activity (apart from damaging it) that results in parents admonishing their children that “this is not what it is meant for.” Of course, parents might restrict the behavior of children playing on the dome—after all, certain actions might be dangerous. But such imposed restrictions do not relate to the fact that the exhibited behavior is not in accordance with *the* function of the play element. Thus, whereas conventional playground equipment, embedded in the social practices, almost “dictates” what children should do, van Eyck’s abstract, minimalistic equipment seems to stimulate the creativity of children—it fosters the children to discover all the affordances it provides them.^1^

### Standardization and Aesthetics

Although van Eyck’s playgrounds were site-specific and thus never standardized, the used play elements in and of themselves often were. As mentioned above, van Eyck was very much inspired by the simple, abstract though powerful forms of Brancusi’s sculptures. He too created elementary forms that are generally organized around principles of geometry. For example, although van Eyck placed his jumping stones sometimes in an irregular pattern (or used stones of different heights), he often used identical stones that he placed in a figure-eight pattern (**Figure 2**). Also in the celebrated “climbing” dome, the bars are placed at equal distances from each other (**Figure 3**). These symmetrical patterns certainly contribute to the aesthetic appeal that van Eyck’s play elements have. Although the principles that guide aesthetic judgments are complex, generally symmetry is found to have a positive influence on an object’s visual attractiveness (e.g., Jacobsen and Höfel, 2003), also for children (e.g., Bornstein et al., 1981).

Yet despite the positive effects on the aesthetics, the symmetrical patterns of van Eyck’s play sculptures are likely to have negative effects on the attractiveness of the sculpture as a play element. Over the last decades, several authors have criticized the standardization of playgrounds. The landscape architect Nebelong (2004), for example, stated that equal distances between bars or jumping stones entail that the child “does not have to worry about his movements,” which will not prepare him “for all the knobby and asymmetrical forms he is likely to be confronted with outside the playground and throughout life” (p. 30). Moreover, and as mentioned above, affordances exist by virtue of the relationship between the properties of the environment and the action capabilities of the animal. Whether a gap between two jumping stones is crossable depends on the gap width relative to the jumping capabilities of the child. And, obviously, children vary in their action capabilities.

Van Eyck developed his play elements primarily for children between 4 and 7 years of age (van Lingen and Kollarova, 2016) and was really concerned with creating the proper distances between, for example, the bars in his climbing frames—experimenting with his own children, he aimed to determine the spacing (Strauven, 2002). And, for example, in his climbing arch there are sometimes different distances between the bars (**Figure 2**), allowing children with varying climbing capabilities to play on it. Also, in the climbing mountains there are different stepping heights (**Figure 3**). However, in many other play elements like the above-mentioned jumping stones and the dome, the distances tend to be equal, rendering the elements mainly interesting for children with matching action capabilities. One might argue that this is not problematic. Also in the City Orphanage, van Eyck created places for different age groups (e.g., Beek and de Wit, 1993). In like fashion, one can conceive of his play sculptures as elements designed for children with certain action capabilities.

However, a recent empirical study by Sporrel et al. (unpublished) casts further doubt upon the standardization of playgrounds. These authors were inspired by an earlier study that revealed that children created varying distances between jumping stones if they were the architect of their own playground (Jongeneel et al., 2015). To test whether children are more attracted to such non-standardized configurations than to the symmetrical configurations that van Eyck tended to design, Sporrel et al. (unpublished) placed both configurations in a public park. The standardized configuration consisted of nine equal-sized stones that were symmetrically organized within the form of a square. The non-standardized configuration, on the other hand, consisted of nine stones of different diameters and heights that were placed at varying distances from each other. Sporrel et al. (unpublished) let children play freely on the configurations and observed that children spent more time playing on the non-standardized configuration than on the standardized, symmetrical one. Moreover, when the children were asked to rate how much they enjoyed playing on the configurations, they reported that they liked the non-standardized configuration better. In fact, messy structures with a fair amount of variation in heights and distances afford children to cross over different gaps. And such an affordance might be an indispensable ingredient of genuine play.

Interestingly, and contrary to the above-mentioned studies on aesthetics, Sporrel et al. (unpublished) also observed that the children reported that they found the non-standardized configuration slightly more beautiful than the standardized one. This seems to suggest that the principles underlying the aesthetic judgments are different when children were to look at objects (as in most studies on aesthetics) than when they were to play on them. What is even more interesting, though, is that Sporrel et al. (unpublished) found no correlation between the children’s aesthetic judgments and their reported joy of play. Apparently, there is no relationship between how beautiful the child found a configuration and how much she enjoyed playing on it. This suggests that although designers might be concerned with the aesthetics of their play elements, the perceived aesthetic is not of overriding importance for the children who play on them.

## Concluding Remarks

In the present paper we have discussed the playgrounds of Aldo van Eyck. As we have seen, these playgrounds not only afforded children to play in the city of Amsterdam after World War II (and stimulated community life), they were also of great architectural significance (e.g., Lefaivre and Tzonis, 1999; Solomon, 2005). Moreover, the abstract play equipment that van Eyck designed was greatly honored by different academic disciplines (e.g., Lefaivre and Tzonis, 1999; Fuchs, 2002; Strauven, 2002; Solomon, 2005, 2014; Sennett, 2008; van Lingen and Kollarova, 2016).

To evaluate the play sculptures of van Eyck, we adopted an ecological approach. This approach aims to understand how animals, including human-beings, regulate their behavior with respect to the affordances of their environments. The concept of affordances has already proved its mettle in the field of architecture (e.g., Beek and de Wit, 1993; Rietveld and Rietveld, 2011; Rietveld, 2016), and hopefully also showed its value in the present paper. We have argued that a sociocultural perspective on affordances can elucidate the insight that the abstract forms of van Eyck’s play elements simulate the creativity of children, an idea that has been forwarded by theorists of art and architecture (e.g., Fuchs, 2002; Strauven, 2002; van Lingen and Kollarova, 2016). The affordance perspective, on the other hand, also revealed a drawback of van Eyck’s play equipment. The symmetry that characterizes many of his play sculptures might be appealing when *only looking at* them, but it seems to reduce the attractiveness of the sculptures as elements for play. However, the studies to date (including this one) count only as a first exploration of the interrelationship between aesthetics, play, and affordances, and much empirical and theoretical work is needed to further scrutinize this.

## Author Contributions

RW and SC: conception of the work, drafting the work.

## Conflict of Interest Statement

The authors declare that the research was conducted in the absence of any commercial or financial relationships that could be construed as a potential conflict of interest.

## Footnotes

1 A reviewer suggested that the open function of van Eyck’s play element might also have a potential drawback—it might restrict the possibility of pretend play (e.g., this pen is now a gun). After all, such play is facilitated by objects having a designated function.
